# Severe Pancytopenia Induced by Valproic Acid

**DOI:** 10.7759/cureus.11252

**Published:** 2020-10-30

**Authors:** Andrew Wahba, Emmalee Bergez

**Affiliations:** 1 Pediatrics, McGovern Medical School, University of Texas Health Science Center at Houston, Houston, USA

**Keywords:** bone marrow suppression, pancytopenia, valproic acid

## Abstract

Valproic acid is commonly used to treat pediatric epilepsy. This drug is usually well-tolerated; its side effects are typically mild, with hepatotoxicity being the most widely recognized one. Bone marrow suppression is a rarely seen complication in patients with valproic acid levels more than 125 mcg/mL. Reported cases indicate an increased incidence of hematologic toxicity; however, evidence for management is limited. We report a case of bone marrow suppression induced by a high dose of valproic acid in a 10-year-old male.

## Introduction

Valproic acid (VPA) is the most commonly used anticonvulsant, initially approved by the U.S. Food and Drug Administration (FDA) in 1978 to be used as a monotherapy or adjunctive therapy for complex partial and absence seizures. Recently, it has been approved for use in bipolar disorder and migraine prophylaxis. VPA is a small-branched chain fatty acid that is highly protein-bound (>85%) in a saturable manner, which results in lower clearance rate and urine excretion. The percentage of protein binding decreases with higher VPA levels. VPA decreases neuronal hyperexcitability through several mechanisms. These include increasing the inhibitory neurotransmitter gamma-aminobutyric acid (GABA) by inhibiting its degradation and increasing synthesis, blockage of the calcium channel voltage-dependent, inhibiting the synthesis of inositol, and modulating glutamatergic transmission [1,2]. VPA is mainly metabolized in the liver through microsomal glucuronidation, which becomes fully effective by the age of four years, mitochondrial beta-oxidation, and, to a lesser extent, cytochrome P450-dependent oxidation [3]. It has an average elimination half-life of 4-16 hours depending on patient age and combined use with enzyme-inducing agents such as phenytoin, carbamazepine, and barbiturates [4,5]. Plasma clearance is 50% higher in the age of 2-10 years compared to adults [6]. VPA has a wide therapeutic trough level concentration between 50 and 100 mcg/mL for seizures and 125 mcg/mL for manic episodes in bipolar disease. Therapeutic daily dose ranges between 15 and 60 mg/kg. VPA is usually well-tolerated; its side effects are typically mild, with hepatotoxicity being the most widely recognized one. Hepatotoxicity usually happens during the first six months of treatment; children under the age of 2 years are at higher risk of developing fatal hepatotoxicity. Other known side effects are pancreatitis, hyperammonemia, hypothermia, suicidal ideations, and birth defects particularly neural tube defects. However, as levels exceed 125 mcg/mL, few cases of hematologic toxicity such as thrombocytopenia, platelet dysfunction, neutropenia, pure red cell aplasia, and acute leukemia have been reported. In this report, we identify a case of severe pancytopenia induced by VPA in a pediatric patient.

## Case presentation

An 11-year-old Hispanic male with a history of autism spectrum disorder (ASD), Dravet syndrome due to *SCN1A* gene mutation, and intractable epilepsy presented with five days of lethargy, decreased oral intake, and seven pounds weight loss. The patient’s mother denied any fever, abdominal pain, vomiting, headache, or sick contacts. His last seizure was six months prior. He previously failed multiple anti-seizure medications such as levetiracetam and clobazam. He had a vagus nerve stimulator placed at the age of five years. He was developmentally delayed with speech and learning difficulties. He had no past medical history of any hematologic disorders. Family history was negative for seizures, developmental, or bleeding disorders. The patient was taking VPA 31 mg/kg/day and lacosamide 7 mg/kg/day for seizure control. He had been on this regimen for the past eight years without any recent new medications or changes.

On physical examination, oral temperature was 98.1°F, heart rate was 85 beats per minute, blood pressure was 97/60 mmHg, respiratory rate was 20 breaths per minute, and oxygen saturation was 99% on room air. Weight was 28.9 kg, with a body mass index (BMI) of 16.59 kg/m^2^. The abdomen was soft, non-distended, non-tender with no hepatosplenomegaly. Heart and lung exams were clear, and no skin rash or lesions were seen. On a neurological exam, he was awake and followed commands, and was able to move all extremities with intact cranial nerves, sensations, and reflexes. No jaundice, oral ulcers, thrush, lymphadenopathy, petechiae, ecchymosis, or signs of bleeding were present.

In the emergency room, lab workup was notable for pancytopenia (Table 1), with a VPA level of 255 mcg/mL (therapeutic range: 50-125 mcg/mL). This VPA level was drawn approximately three hours after the last dose was given. VPA was discontinued, topiramate was initiated with lacosamide for proper seizure prophylaxis, and the patient was admitted for further workup of pancytopenia. Reticulocyte count on admission was 1.5 %, demonstrating inadequate bone marrow response to pancytopenia. On the following day, the patient developed conjunctival pallor and bilateral lower extremities petechiae. Vital signs remained within normal limits. On repeat lab tests, the patient had worsening pancytopenia (Table 1), with an absolute neutrophil count (ANC) of 300/mm^3^. Due to concerns for altered mental status in the setting of severe thrombocytopenia, a CT of the head was obtained, which showed no signs of intracranial bleeding. The peripheral smear showed evidence of thrombocytopenia and leukopenia, but no blasts or other concerns for leukemia were identified.

**Table 1:** Laboratory values at different time points WBC, white blood cell; RBC, red blood cell; MPV, mean platelet volume; ALT, alanine aminotransferase; AST, aspartate aminotransferase; BUN, blood urea nitrogen

Lab Test	Day 1	Day 2	Day 4	Day 5	Day 6	Day 8	Day 9
Valproic acid (mcg/mL)	255	214	102	53	31	6	
WBC x 10^3^/mm^3^	5.1	3.6	3.6	2.3	4.9	6.8	7
RBC x 10^6^/mm^3^	3	2.2	2.2	1.7	3.2	3.2	3.1
Hemoglobin (g/dL)	10.7	7.9	7.9	6.4	11.2	11.3	11
Hematocrit (%)	31.9	22.6	23.1	18.4	32.5	33.2	31.1
Platelet x 10^3^/mm^3^	10	4	7	3	124	103	143
MPV (fL)	9.7	9.4	9.2	8.2	7.8	9.4	9.5
Neutrophil x 10^3^/mm^3^	0.9	0.3	0.7	0.4	1.7	1.6	1.5
Monocytes x 10^3^/mm^3^	0.3	0.1	0.4	0.8	1.4	1.6	1.2
Lymphocyte x 10^3^/mm^3^	3.9	3.2	2.5	1.6	2.3	3.6	4.2
Reticulocyte count (%)	1.5				3.6	5.4	4.7
ALT (unit/L)		12			17	25	
AST (unit/L)		22			45	64	
Ammonia (μg/dL)		147	103	79	66	78	74
Total bilirubin (mg/dL)		0.8			0.7	0.5	
Albumin (g/dL)		3			2.9	2.9	
Sodium (mEq/L)	140	141			140	139	
Potassium (mEq/L)	3.7	3.9			4.2	4	
Creatinine (mg/dL)	0.50	0.43			0.42	0.39	
BUN (mg/dL)	15	13				16	

The underlying reason for elevated VPA levels was unclear despite the patient's medication compliance. Since VPA is mainly metabolized in the liver, factor V level was obtained to assess liver function, which returned within normal limits, and abdominal ultrasound showed normal size liver with normal echogenicity. The patient had previous outpatient liver enzyme levels that were within normal limits, the most recent of which was six months prior.

A trial of intravenous immunoglobulin (IVIG) 1 g/kg was given on admission day 3 to determine if the process was immune-mediated. However, follow-up laboratory values continued to show pancytopenia despite the VPA level progressively decreasing to 102 mcg/mL. To rule out other causes of bone marrow aplasia, Epstein-Barr virus (EBV), cytomegalovirus (CMV), human immunodeficiency virus (HIV), and parvovirus B19 serology were negative. He had normal vitamin B12, erythrocyte sedimentation rate (ESR), lactate dehydrogenase (LDH), haptoglobin, and D-dimer levels. Neutrophil antibody was undetected by flow cytometry.

Due to persistent anemia and severe thrombocytopenia, a single transfusion of 10 mL/kg packed red blood cells and 10 mL/kg platelets were given for supportive measures on admission day 5. On the following days, the patient’s laboratory values showed evidence of gradual bone marrow recovery, and the patient was discharged on day 9.

## Discussion

We report a case of a pediatric patient with a history of intractable epilepsy and *SCN1A* mutation causing Dravet syndrome who has been receiving VPA for eight years with no reported side effects. He presented with toxic VPA levels and severe pancytopenia. VPA affected all the three bone marrow cell lines, which started to recover around day 6 after discontinuation of the drug.

*SCN1A* gene, located on chromosome 2q24, is one of the most commonly known epilepsy genes. Our patient has a missense variant c.1096G>C; p.Asp366His (one guanine ribonucleotide was altered to cytosine in codon 1096, which caused a change in the reading frame from aspartate to histidine). *SCN1A* mutations have been linked to multiple epilepsy disorders such as Dravet syndrome, which is also known as severe myoclonic epilepsy in infancy (MIM# 607208). Dravet syndrome usually presents as a refractory seizure during the first year of life and developmental delay. *SCN1A* mutations impair the inhibition of neuronal sodium channel activity, which increases neuronal excitability [7,8].

Our patient had severe pancytopenia, hyperammonemia with normal liver enzymes, and kidney function levels. L-carnitine has been shown to be effective in VPA-induced hepatotoxicity and hyperammonemia above 80 μg/dL [9]. Exogenous carnitine binds to VPA, enhancing the beta-oxidation and urea synthesis process, thus decreasing ammonia levels. Since the repeat ammonia level for our patient dropped below 80 μg/dL, the decision was to trend the ammonia level without giving carnitine.

Upon admission, differential diagnoses for pancytopenia included drug-induced myelosuppression, autoimmune-mediated pancytopenia, and other causes of bone marrow failure such as idiopathic acquired aplastic anemia, hypoplastic myelodysplastic syndrome, infections, nutritional deficiencies, hematopoietic and lymphoid neoplasms, and myelofibrosis. Peripheral smear findings, negative viral serology, negative neutrophil antibody, poor response to IVIG, and spontaneous recovery after discontinuation of VPA support that the cause was most likely drug-induced bone marrow suppression.

VPA can cause a wide spectrum of hematologic toxicities such as thrombocytopenia, acquired Von Willebrand disease, neutropenia, Pelger-Huet anomalies, macrocytosis, pure red cell aplasia, and acute leukemia. These toxicities appear to be reversible and dose-related with the female gender being at higher risk [10,11]. Mild thrombocytopenia is the most commonly reported toxicity, and it usually does not require immediate discontinuation of the drug [12]. Few cases of severe pancytopenia have been reported. Direct bone marrow suppression and immune-mediated destruction are the two known mechanisms. In an in vitro study to assess direct bone marrow suppression, VPA markedly inhibited the growth of bone marrow progenitor cells at a concentration of 200 mcg/mL, and normal growth was seen at a concentration of 100 mcg/mL [13]. We believe the patient's supra-therapeutic VPA levels have led to the severity of his symptoms. Management is mainly supportive through discontinuing VPA in severe cases or lowering the daily dose in mild cases, use of IVIG in immune-mediated toxicity, desmopressin or aminocaproic acid in patients with bleeding diathesis, granulocyte colony-stimulating factor (GCSF) in neutropenic patients, and blood transfusion in life-threatening conditions [14,15].

## Conclusions

VPA-induced hematologic toxicity is becoming more frequently encountered; however, evidence for management is limited. Bone marrow suppression is a rarely seen complication in patients with VPA levels > 125 mcg/mL. In these patients, immediate discontinuation of the drug is recommended. Resolution of the bone marrow suppression is expected to occur within 10 days of VPA withdrawal. Patients taking VPA may benefit from periodic monitoring of cell line counts, and caution should be taken when prescribing high doses.
